# Reliable scaling of position weight matrices for binding strength comparisons between transcription factors

**DOI:** 10.1186/s12859-015-0666-1

**Published:** 2015-08-20

**Authors:** Xiaoyan Ma, Daphne Ezer, Carmen Navarro, Boris Adryan

**Affiliations:** 1 Department of Genetics, University of Cambridge, Downing Street, Cambridge, CB2 3EH UK; 2 Cambridge Systems Biology Center, University of Cambridge, Tennis Court Road, Cambridge, CB2 1QR UK; 3 Department of Computer Science and Artificial Intelligence, University of Granada, Periodista Daniel Saucedo Aranda, Granada, Spain

**Keywords:** Transcription factor, Position weight matrix (Position-Specific Scoring Matrix), Binding site strength

## Abstract

**Background:**

Scoring DNA sequences against Position Weight Matrices (PWMs) is a widely adopted method to identify putative transcription factor binding sites. While common bioinformatics tools produce scores that can reflect the binding strength between a specific transcription factor and the DNA, these scores are not directly comparable between different transcription factors. Other methods, including p-value associated approaches (Touzet H, Varré J-S. Efficient and accurate p-value computation for position weight matrices. Algorithms Mol Biol. 2007;2(1510.1186):1748–7188), provide more rigorous ways to identify potential binding sites, but their results are difficult to interpret in terms of binding energy, which is essential for the modeling of transcription factor binding dynamics and enhancer activities.

**Results:**

Here, we provide two different ways to find the scaling parameter *λ* that allows us to infer binding energy from a PWM score. The first approach uses a PWM and background genomic sequence as input to estimate *λ* for a specific transcription factor, which we applied to show that *λ* distributions for different transcription factor families correspond with their DNA binding properties. Our second method can reliably convert *λ* between different PWMs of the same transcription factor, which allows us to directly compare PWMs that were generated by different approaches.

**Conclusion:**

These two approaches provide computationally efficient ways to scale PWM scores and estimate the strength of transcription factor binding sites in quantitative studies of binding dynamics. Their results are consistent with each other and previous reports in most of cases.

**Electronic supplementary material:**

The online version of this article (doi:10.1186/s12859-015-0666-1) contains supplementary material, which is available to authorized users.

## Background

Sequence-specific transcription factors (TFs) are key elements in the regulation of gene expression. Their binding preferences to DNA have been studied extensively *in vitro*, *in vivo* and using computational methods. *In vitro* methods such as protein binding microarray(PBM) [1], high-throughput SELEX measurements [2] and DNase I-seq [3] have provided fundamental insight into the specificity of TF binding. The systematic compilation of DNA sequences from such experiments (and along with them catalogues such as TRANSFAC [4] or JASPAR [5]) have long suggested that TFs do not just bind to one DNA motif, but can bind to a repertoire of similar sequences. Stacks of such sequences give rise to alignment matrices, in which each column represents the absolute count of A, C, G and T nucleotide occurrences per position along the length of the motif. We use “motif” in this manuscript in reference to the PWM motif for a specific TF. Work by Berg et al. [6] introduced a derivative of the alignment or position frequency matrix (PFM), the position weight matrix (PWM, sometimes also noted as PSSM for position-specific scoring matrices), which takes the log likelihood of observing nucleotides taking their overall frequency into account. Berg et al. [7] later showed that the score obtained by comparing the PWM against a DNA sequence is proportional to the binding energy between this TF and the DNA. In most cases the actual binding energy between the protein and DNA is not known, and the proportionality is scaled with a factor commonly termed *λ*. Berg et al. originally introduced *λ* to relate the population of base-pair choices to binding free energy [6]. It is analogy to the inverse temperature factor in statistical physics to describe the energy distribution and also to serve as a factor in tuning the number of potential binding sites in order to satisfy the constraints on overall energy distribution.

There is no well-characterized and easily computable way to determine the TF binding energy for specific DNA sequences and to compare binding site strength between different types of TFs at large scale. This is problematic when scanning the genome with a library of PWMs, as scoring functions treat each PWM independently, and the absolute score associated with a “good match” to the PWM of one transcription factor might be associated with a mismatch for another factor. A more sophisticated application of binding site strength estimation is, for example, modeling the relationship between enhancer occupancy and gene expression [8, 9]. The experimental PBM approach [1] allows the estimation of the relative binding strength of a protein to “naked” DNA *in vitro*, but the data availability is restricted to a limited number of TFs due to high cost of the technology. In addition, PBMs are also not suitable for TFs with longer motifs, as their accuracy will decrease with the length of the DNA probe [1]. Therefore, PWM-based approaches are used to computationally estimate TF binding affinity to a specific sequence [8, 10].

In the majority of bioinformatic studies, the scaling factor *λ* is unknown and PWM scores are used at face value as measure of affinity. For example, in our own work [11] we used the PWM score without scaling to compare binding site strength across different TFs in *E. coli*, which might lead to a bias due to the absolute differences between the highest and lowest PWM scores across all TFs of interest. One approach is to scale the PWM score by a p-value for each specific score threshold [12]. This method provides a good way to define putative binding sites by choosing a proper statistical threshold, but it is difficult to correlate these p-values with binding energy estimation, as is required for quantitative studies of enhancer activity [8, 9]. Other work has tried to assess the range of *λ* on the basis of fitting calculated affinity landscapes to ChIP-seq profiles [13, 14]. However, ChIP data is intrinsically noisy and the height of a ChIP peak may not accurately represent the real binding affinity, undermining the stability and accuracy of *λ* obtained from these methods. In Roider et al. [13], the estimated *λ* for the same TF in different conditions diverged greatly in nearly one third of TFs. Furthermore, there is a wide band of possible *λ* values that optimize the correlation. Aforementioned fitting methods are further reliant on chromatin accessibility data acquired under the same growing conditions or development stages, which is sometimes not available for specific TFs.

We propose a simple approximation to estimate the scaling parameter *λ* based on existing PWMs, average maximum mismatch energy tolerance estimated by high-throughput binding energy measurements [15] and the distribution of PWM scores across the genome of a specific organism. This method is independent of genome-wide binding and accessibility data. Furthermore, in the cases where there are potentially inconsistent PWMs for a particular TF (*e.g.* derived on the basis of individual binding sites vs. derived from high-throughput efforts), we provide a method to convert the known *λ* for one PWM of the same TF into another suitable value for a new PWM. This method is based on a computational model of the facilitated diffusion of TFs on the DNA that our group established earlier [16]. We calculate sequence-specific residence times of TFs at the DNA, which is correlated with affinity. We can therefore derive *λ* for different PWMs of the same TF on the basis of the consistency of simulated residence time. These two strategies (a) calculating *λ* to scale PWM scores based on the mismatch energy theory using a simple equation and (b) converting the scaling parameter *λ* between different PWMs of the same TF on the basis of simulated residence time of facilitated diffusion provide simple but useful estimations of binding energy across different TFs using properly scaled PWM scores.

## Methods

### PWMs of TFs for yeast, fly and vertebrates

Position frequency matrices (PFM) used to construct PWMs were downloaded from the JASPAR database (JASPAR-CORE-2014 non-redundant PFM) [5]. Additional sources of PFMs such as those contained in the BioConductor package *PWMEnrich.Dmelanogaster.background* [17] were used as a source of different matrices for the same TFs. PFMs constructed with less than 30 reference sequences of validated binding sites were removed, as we deemed those insufficient descriptions of binding preference. Given that typical TF binding sites span at least six base pairs, we removed any motifs less than 6 base pairs in length.

A bioinformatics approach was used to derive PWM scores [18] as follows:

(1) $$ S_{j}=\sum_{k=1}^{L}{\log_{2}{\frac{v_{j,k}}{f_{j+k}}}}  $$

where *j* is the DNA position for the PWM score calculation, *L* is the length of the motif and *k* represents *k* ^*th*^ nucleotide in the PWM motif. In addition, if there is a specific nucleotide in position (*j*+*k*) on the DNA, *f*_*j*+*k*_ is the frequency of this nucleotide in the whole genome of a specific organism. Nucleotide frequency used for this study in each organism were as follows: *D. melanogaster*: 0.28 for A and T, 0.22 for G and C; *S. cerevisiae*: 0.31 for A and T, 0.19 for C and G; vertebrate including human and mouse: 0.29 for A and T, 0.21 for C and G. Please note that the choice of background frequencies can be critical, and that adjustments to local extrema may be necessary. We used a pseudo-count *μ* to adjust the frequency of nucleotides and obtain *v*_*j*,*k*_ to avoid zero frequency as follows [19]

(2) $$ v_{j,k}=\frac{n_{j,k}+f_{j+k}\cdot\mu}{\sum_{x}n_{x,k}+\mu}  $$

where *μ* is chosen to be 1 [19] and we also show that the choice of the pseudo-count *μ* does not have significant influence on our results (Additional file 1: Figure S6); *n*_*x*,*k*_ is the frequency of certain nucleotide *x* in a specific position *k* of the motif.

### Simple equation to calculate *λ*

*λ* is the scaling factor that allows for direct comparison of different PWMs in terms of binding energy to DNA. Based on the mismatch energy theory for estimating TF binding strength [7], the mismatch energy at a particular binding site *j* of TF species *i* in the genome can be expressed as:

(3) $$ E_{mismatch,i,j} ={\Delta}S_{i,j}/{\lambda_{i}} =(S_{max,i}-S_{i,j})/{\lambda_{i}}  $$

where *S*_*i*,*j*_ stands for the PWM score at position *j*, *S*_*max*,*i*_ is for the maximum PWM score of TF species *i* and *λ*_*i*_ is the scaling parameter we want to estimate. Note that the mismatch energy we refer to in the text is derived from information theory, with the unit of bits, which can also be described as “mismatch bits”. This is useful in a variety of contexts, such as comparing the binding strength of different TFs. In addition, the expected amount of time that the TF is bound to a particular DNA sequence can be estimated as:

(4) $$ {\tau}_{j}={\tau}_{0}({\lambda})\cdot e^{-S_{j}/{\lambda}}  $$

where *S*_*j*_ is the PWM score at position *j* in the genome, *τ*_0_ is the average residence time calculated as in [16]. This equation is widely used in simulations of TF binding kinetics [20].

Given the utility of the *λ* for estimating binding strength and occupancy time, it is very important to have a simple strategy for estimating it. We derive our equation based on the following core assumptions: 1) The top 0.1 % of the highest scoring matches of the PWM to intergenic regions are considered to be possible TF binding sites, as suggested by [21]. Their genome-wide study of different eukaryotic TFs revealed an average of 1 binding site in every 1-5 thousand base pairs of intergenic sequence. This top 0.1 % threshold has also been similarly adopted in other studies [10]. In addition, if varying this threshold from top 0.01 % to top 1 ·10^−4^ and 1 ·10^−5^, the rank of calculated *λ* still shows good correlation in each group of organisms(Additional file 2: Figure S5). 2) The maximum mismatch energy between the consensus binding motif and specific DNA sequences is proportional to the information content of the PWM matrix of the TF. Note that the mismatch energy we refer to in the text is derived from information theory, with the unit of bits, which can also be described as “mismatch bits”. The information content (*If*) of the PWM matrix is defined below [7],

(5) $$ If=\sum_{k=1}^{L}\sum_{i \in A,T,C,G}{ p_{i,k}{\log_{2}{\frac{p_{i,k}} {f_{i}}}}}  $$

where *k* is the *k* ^*th*^ nucleotide in the PWM motif, *f*_*i*_ is the background nucleotide frequency, and *p*_*i*,*k*_ is the adjusted frequency of nucleotide *i* in position *k* which is defined as follows,

$$p_{i,k}=\frac{{nu}_{i,k}+f_{i} \cdot\mu}{\sum_{i}{nu}_{i,k}+\mu} $$

where *n* *u*_*i*,*k*_ is the frequency of certain nucleotide *i* in a specific position *k* of the motif, and *f*_*i*_ is the background nucleotide frequency.

The lower boundary of potential binding sites is approximated by the top 0.1 % of PWM scores following the same reason as mentioned before and corresponds to the maximum mismatch energy tolerance level as follows:

$$E_{maxMismatch,i}=\frac{S_{max,i}-S_{top0.1~\%,i}}{\lambda_{i}} $$

where *E*_*maxMismatch*,*i*_ stands for maximum mismatch energy tolerance for TF species i, thus, *λ*_*i*_ can be calculated using:

(6) $$ {\lambda_{i}}=\frac{S_{max,i}-S_{top0.1~\%,i}} {E_{maxMismatch,i}}  $$

Because different transcription factors have different DNA binding domains, the maximum mismatch energy range can vary from one TF to another. Since there is only data available for 4 individual TFs using microfluidic platform-based binding energy measurements [15], we estimated the maximum mismatch energy for other TFs by using the available data as the average rate and assuming that the mismatch energy tolerance is proportional to the information content of the PWM as follows:

(7) $$ E_{maxMismatch,i}=<E_{maxMismatch}>\times \frac{{If}_{i}}{<If>}  $$

where <*E*_*maxMismatch*_> stands for the average maximum mismatch energy tolerance, which is chosen to be 6 bits as is discussed below from the study of Maerkl et al. [15]; *I* *f*_*i*_ represents the information content of a specific PWM and <*I* *f*> stands for the average information content corresponding to the average maximum mismatch energy [15], which is 13.2 bits. We reason that if the information content is a good indication of how specific a TF is, the energy drop measured in bits between strong and weak binding sites (*S*_*max*,*i*_−*S*_*top*0.1 *%*,*i*_)/*λ*_*i*_ should have some relationship with the binding specificity of a particular TF. The more specific a TF is, the more significant the energy drop can be. Given limited data in binding energy measurement, we assume that the relationship is simply linear.

We chose an average mismatch energy tolerance of 6 bits based on the study by Maerkl et al. 2007 [15]. They showed by mechanical trapping of molecular interactions a significant decline in binding energy by at most 2 to 3 nucleotide mismatches, and each mismatch nucleotide contributes 2 bits in mismatch energy. Even if more mutations are introduced, the binding energy does not drop further since it has already reached the background non-specific binding energy level.

This experiment was applied only to TFs belonging to the bHLH family. In the absence of more comprehensive data, we must assume that all TFs share this value; although if more general TF in-vitro binding energy measurement results become available, we suggest adjusting the specific top score threshold and corresponding average mismatch energy bits accordingly. Another report featuring TFs from different families including: p53, Max, Glucocorticoid Receptor [22] also provides additional support for 6 bits as average mismatch energy tolerance level since TFs from different families in their study have similar binding kinetics.

In order to control for PWM motif length, in the analysis of *λ* value comparison across different species and TF families, each *λ* value was transformed into a Z-score. Specifically, PWM motifs were grouped by motif length, with each group having more than 50 PWM motifs (The groups were: 7-8 bp, 9-10 bp, 11-12 bp, 13-15 bp, >=16 bp), and the *λ* values were normalized by the mean and standard deviation within each of these groups (Additional file 3: Table S3 lists the mean and standard deviation value for each group, Additional file 4: Figure S3 depicts the distribution of *λ* at different motif lengths with color coded points that represent different species).

### Estimating *λ* of a new PWM matrix for the same TF based on the residence time landscape of the facilitated diffusion model

Sometimes there may be more than one PWM available for a specific TF. For instance, different TF motif databases (such as JASPAR [5], SwissRegulon [23], FlyFactorSurvey [24], and HOCOMOCO [25]) may have different versions of PWM motifs for the same TF. In order to directly compare the TF binding energy when using two alternative versions of a PWM, it is important to have a way of scaling the results by *λ*. *λ* can be adjusted using the formalism introduced in the previous sections. As a compute-efficient alternative, we developed a more optimal strategy for estimating *λ*, which does not require the assumption that the PWM information content influences the energy mismatch tolerance. Instead, we base our strategy on the estimation of the sequence specific residence time of a particular TF, which is a biologically meaningful quantity and can be correlated with *in vitro* sequence-dependent sliding measurement of TFs [10]. For the same TF, the distribution of the sequence-specific residence time calculated by Eq. 4 should be as consistent as possible, even when using slightly different PWMs if an appropriate *λ* is chosen for scaling. Based on this, given a known *λ* for one PWM, we are able to find another suitable *λ* for the new PWM.

Note that the stronger the PWM score, the more likely it is that the sequence is bound by a TF and that the residence time of a TF is a biologically meaningful quantity, but there is a much greater number of weak and medium strength binding sites than there are strong sites in the genome. Therefore, if we scored each potential binding site equally, the background of weak and medium strength binding sites would have a greater affect on the estimated *λ* than the strong binding sites. Therefore, we compare residence times across different quantiles on a logarithmic binding strength scale so that the strongest binding sites have the most influence on our *λ* estimates.

Specifically, in the following analysis, we take the −*l* *o* *g*_10_ of the cumulative distribution of PWM scores and select all binding sites with values greater than 3.0 (recall that this corresponds to the 0.1 % percent of binding sites, which were chosen as the lower boundary of weak binding sites). We divide these top-scoring binding sites into bins every 0.1 log-quantile and calculate the average residence time for each of these bins. Our strategy identifies the *λ* that would produce the most similar residence times for each of these log-quantiles. Assuming that for the first PWM we already have an estimate of *λ*, by either binding profile fitting or other methods, we can use Eq. 4 to calculate the residence time for each binding strength log-quantile, as described above. In the following analysis of this paper, we borrow the values obtained from Eq. 6 as pre-calculated *λ* for proof-of-principle, since there are very few well-characterized *λ* values from profile fitting. Note that *τ*_0_ is calculated via the strategy described in Zabet et al. [16] from all intergenetic regions in the genome, which has a different value for each unique PWM.

Now for the second PWM, we can vary *λ* between the potential values of 0.1 and 3, which was shown to be a possible *λ* range [13], and calculate the corresponding residence times at each log-quantile level. We can now compare the reference residence times from the first PWM with the residence times for the second PWM across each binding site strength level, and for each value of *λ*. The *λ* that minimizes the mean square error between two sets of calculated residence times is chosen as the suitable *λ* value for the second PWM matrix. Since outliers can have a big influence on the mean square error, we calculated the sum of the absolute differences for the natural logarithm of residence times between the two PWM matrices for these quartile bins (Eq. 8) to make a comparison with the method that uses mean square error.

(8) $$ {\sum\nolimits}_{q}|\ln{{\tau}_{q,{\lambda}}}-\ln{{\tau}_{q,ref}}|  $$

where *q* represents each quantile in the quantile series, *τ*_*q*,*λ*_ is the residence time in a specific quantile of a particular *λ*, *τ*_*q*,*ref*_ is the residence time in the same quantile of the known *λ* of the reference PWM matrix. The *λ* derived by minimizing the mean square error or minimizing the value of the above formula show good consistency with adjusted *R* ^2^ of 0.9644 (p= 6.3·10^−9^). Thus, there should not be significant bias using either of these two methods.

The R scripts for both converting *λ* between two PWM matrices and estimating *λ* using Eq. 6 are provided in the following link: https://github.com/XyMa/PWM_scale.

## Results

### Estimating scaling parameter *λ* for binding site affinity across different species and TF families based on Eq. 6

The *λ* parameter is the critical link between PWM score, the estimated binding energy and TF residence time. Estimating TF binding site affinity by comparing PWM scores at face value can lead to a large bias, especially when this includes comparisons between many types of TFs, because several properties of the PWM itself can influence the PWM score. For example, the information content of the PWMs is positively correlated to the maximum possible PWM score, as is shown in Additional file 5: Figure S1 with an *R* ^2^ value of 0.597. Thus, the absolute value of PWM scores cannot be compared directly across different TFs as an indicator of binding site strength. Proper scaling of PWM score is needed in order to compare binding site affinity across different types of TFs. Based on the methods proposed by Berg et al. [7], the TF binding energy for a specific binding site can be computed by Eq. 3 using the estimated *λ*.

**Fig. 1:**
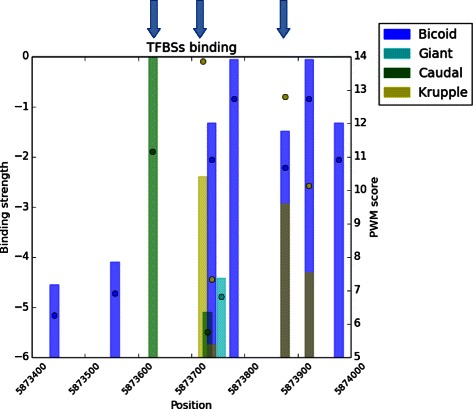
A comparison between PWM score and binding site strength in the *D. melanogaster even-skipped stripe 1* enhancer. The *even-skipped stripe 1* enhancer on chromosome 2R is dense with binding sites. We compare the raw PWM scores (circles) and the *λ*-scaled binding strength (height of the bars) for each of these binding sites, colour-coded by the type of TF. Based on raw PWM scores, one might assume that the Caudal site indicated by the first blue arrow would have a lower binding strength than the Kruppel site indicated by the second blue arrow; Eq. 3 instead of Eq. 5, it becomes evident that the opposite is the more likely scenario. The third arrow points to a location where a Kruppel and a Bicoid binding site overlap. Here, the *λ* adjusted binding strength estimates would suggest that Bicoid binding site is stronger, while a raw PWM score would suggest the opposite. These results illustrate how using raw PWM scores may result in biased interpretation of the relative binding strength of TFs

*λ* calculated by this method are all within the range suggested by Roider et al. [13], which are listed in Table 1 for different organisms. The values for vertebrate species refer to all available vertebrate TFs obtained from the non-redundant PFM JASPAR database. The upper and lower bound of *λ* across all organisms are quite similar, in the range of 0.25 to 2.83. This indicates that all eukaryotic TFs, no matter which organisms they belong to, all share energetically similar DNA binding mechanisms, since *λ* can be interpreted as a metric for the chemical property of stickiness between the TF molecule and DNA. To demonstrate the biological applications of this parameter, Fig. 1 shows an example of the *D. melanogaster Even-skipped stripe 1* enhancer with the comparison between PWM score and the affinity estimation using *λ* scaling. The usefulness of *λ* estimates becomes apparent when comparing the first two binding sites indicated by blue arrows in this locus; the second binding site has a higher PWM score, but its binding strength is lower than the first binding site once the *λ* scaling factor is taken into account. Similar situations also appear in the overlapping binding site of Bicoid and Kruppel indicated by the third arrow. Thus, only comparing the raw value of PWM score [11] may lead to false interpretations of binding site importance. Although there is no current experimental evidence for the relative importance of binding sites for this specific enhancer, this example serves to demonstrate how a different interpretation of the contribution of individual binding sites can lead to alternative testable hypotheses.

**Table 1:** Maximum, minimum and the mean values of *λ* in 3 groups of organisms

	*S. cerevisiae*	*D. melanogaster*	Vertebrates
maximum	2.83	2.72	2.82
minimum	0.26	0.35	0.25
mean	1.25	1.40	1.73

**Fig. 2:**
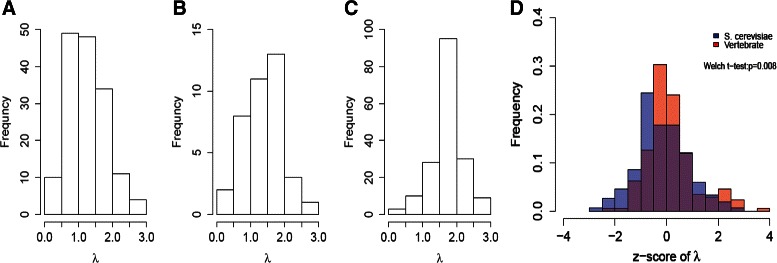
*λ* distributions across difference organisms. The histograms depict the *λ* values estimated from Eq. 6 for the JASPAR non-redundant core motifs in *S. cerevisiae* (**a**), *D. melanogaster* (**b**) and available vertebrates (**c**) [5]. Subfigure D depicts the comparison between z-score distribution of *λ* for vertebrate and yeast TFs after controlling for motif length

**Fig. 3:**
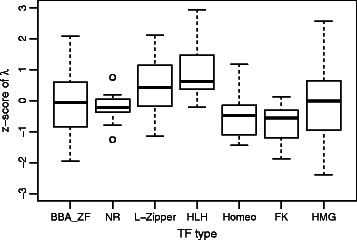
*λ* z-score distribution comparison across major TF families. BBA-ZF represents the *λ* distribution for *β*- *β*- *α* zinc-finger family; NR is zinc-finger nuclear receptor family; L-zipper stands for the basic leucine-zipper family; HLH is helix-loop-helix family; Homeo is homeobox family; FK is fork-head family and HMG is high mobility group family. For each group, *λ* was calculated by Eq. 6 and z-score is obtained by normalizing *λ* in each PWM motif length group

Next, we calculated *λ* for each TF in *S. cerevisiae*, *D. melanogaster* and available vertebrate TFs in JASPAR [5], which are listed in Additional file 6: Table S1. Figure 2 a to 2c show the overall *λ* distribution in each group of organisms. After controlling for motif length, there is a significant difference between vertebrate and *S. cerevisiae* motifs (Welch t-test p-value = 0.008) (Fig. 2 d) and between *D. melanogaster* and vertebrate motifs (p-value = 0.043), but no significant difference between *S. cerevisiae* and *D. melanogaster*. Furthermore, we grouped *λ* values, normalized by PWM motif length, according to different TF families in JASPAR [5] (Fig. 3). The distribution of raw *λ* values across different TF-families are depicted in Additional file 7: Figure S2. The basic leucine-zipper family and helix-loop-helix family are two families with the highest average z-score of *λ*, compared with other groups with Welch t-test p-values equal to 8.9·10^−4^ and 3.7·10^−5^ respectively. TF families that belong to the same superfamily show similar *λ* distribution. For example, *β*- *β*- *α* zinc-finger family and the zinc-finger nuclear receptor family both belong to the zinc-finger TF super family, and no significant difference is detected between these two (Welch t-test p value = 0.35), while both are significantly lower than the aforementioned two families (p-value = 0.012 and 5.0·10^−5^). In addition, homeobox and forkhead TF families, both of which belong to the helix-turn-helix(HTH) TF super family, show no difference in *λ* z-score distribution (p value = 0.27), but appear to have lower average *λ* compared with leucine-zipper, helix-loop-helix family and zinc-finger super family (Welch t-test p-value equals to 5.2·10^−6^, 1.6·10^−7^ and 2.2·10^−4^, respectively).

Since *λ* is the denominator to the PWM score differences between one binding site and the consensus sequence in Eq. 3, a larger *λ* indicates lower mismatch energy when *Δ* *S*_*j*_ is the same. Thus, with the same possible mismatch energy range, if *λ* is larger, the PWM score can have a greater range from the consensus sequence to the potentially weakest binding site, which indicates that, as suggested by Pabo et al. [26], the binding motif for the TF family has higher flexibility. This is consistent with the fact that the TFs in the zinc-finger super-family, including the nuclear receptor and *β*- *β*- *α* zinc-finger families, are less constrained to a particular motif than HTH super family. Additionally, cross species comparison of *λ* indicates that from yeast to vertebrate, more flexible TF motifs are used, which is consistent with the result from Itzkovitz et al. [27] that organisms which appeared more recently in evolution tend to use more TFs with motifs of higher flexibility.

### Comparison of *λ* values estimated with Eq. 6 to *λ* values derived from fitting ChIP-seq data

**Fig. 4:**
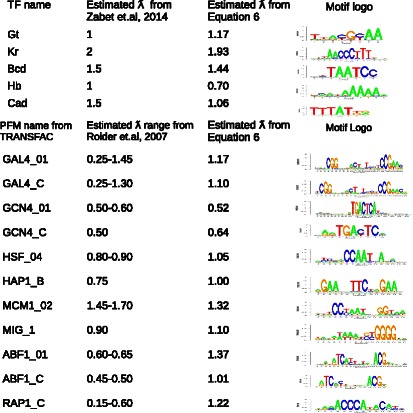
Comparison of *λ* values estimated with Eq. 6 to *λ* values derived from fitting ChIP-seq data of Zabet et al. [14] and Roider et al. [13]

We compared our estimated *λ* values with those estimated from ChIP-seq experiments by Zabet et al. [14] (See Fig. 4). Equation 6 provides a close approximation of all five values estimated in this paper (adjusted *R* ^2^= 0.64, p-value = 0.061). We also compare our results with the *λ* values reported by Roider et al. 2007 [13] for 11 yeast TF motifs from TRANSFAC [4] (See Fig. 4). For each of the 11 TFs, Roider and colleagues fit *λ* values to ChIP-seq data from cells grown in different growth mediums leading to a range of potential *λ* values for each TF. However, for each specific cell growth condition, only the most optimal value of *λ* was selected for each TF, even with circumstances in which there is a plateau in the parameter space with many possible *λ* values fitting the data nearly equivalently. The range of *λ* values from their study and the estimated results from Eq. 6 using default parameters are listed in Fig. 4. Our *λ* value estimations are within, or very close to, their estimated range for 8 out of 11 motifs belonging to 6 out of 8 TFs (absolute differences within 0.25), but another 3 motifs for 2 TFs show poor correlation. It is possible that in some specific cases the assumed default parameters in Eq. 6 could deviate from the real binding properties of these TFs, which can potentially lead to some bias in the estimation of *λ*. Alternatively, these *λ* values might lie within the parameter plateau region, and might be a suitable fit for the experimental data.

### Converting *λ* between different PWM matrices of the same TF

In many cases there are two PWMs available for the same TF, and one of these PWMs might already have a reliable estimate of *λ* from any number of experimental or computational approaches [14]. In such circumstances, we provide a strategy to estimate the unknown *λ* associated with the alternative PWM. It would be possible to calculate the unknown *λ* from Eq. 6, but this does not incorporate the additional data available (i.e. the known *λ*). Our alternative strategy not only incorporates this data, but also loosens the assumption in Eq. 6 that the maximum mismatch energy for DNA binding is proportional to information content.

**Fig. 5:**
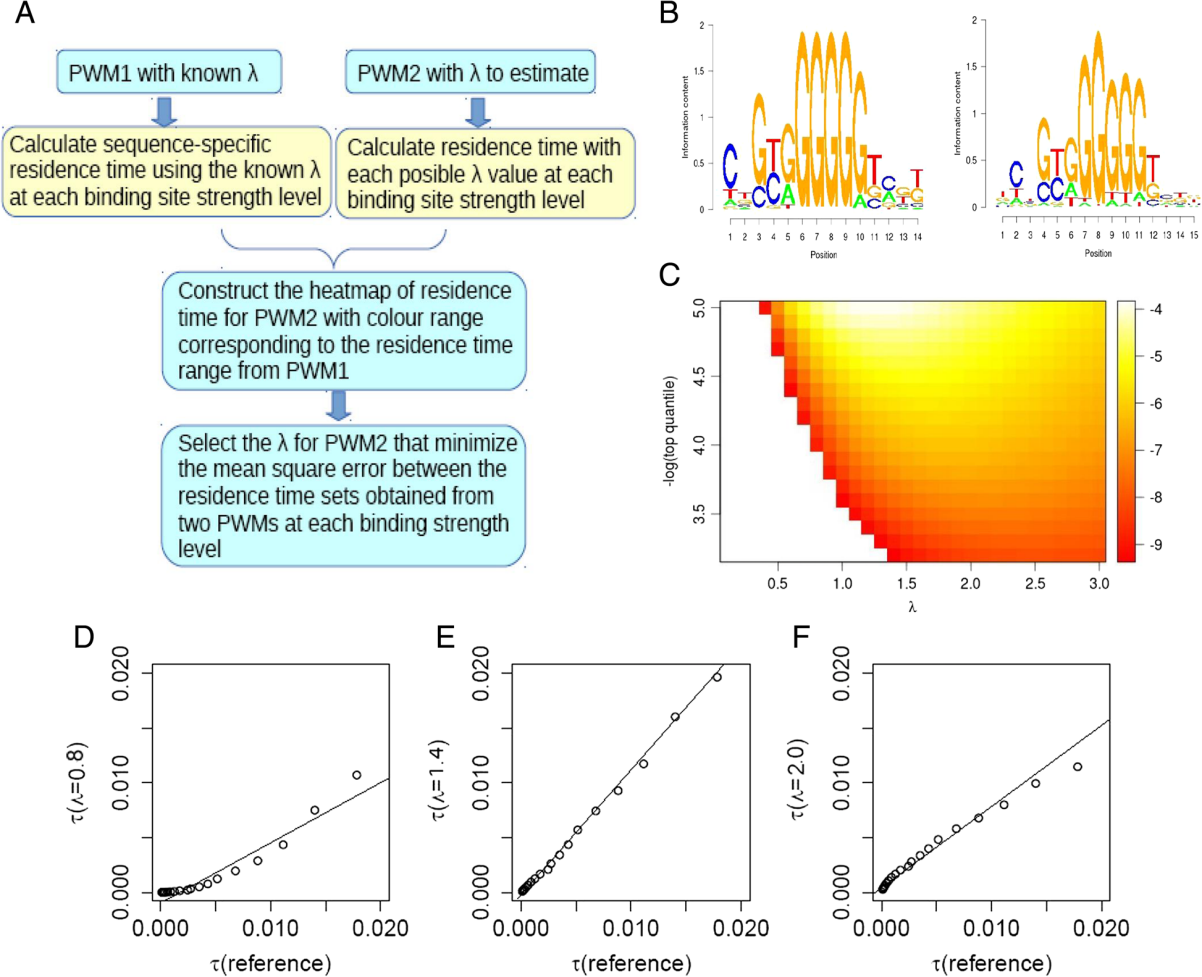
Conversion of *λ* between two PWM matrices for the lmd transcription factor. The flow chart shows the procedure to obtain an optimised *λ*, given two different PWMs and one known and one unknown *λ* (**a**). Subfigure **b** illustrates the two alternate PWMs for lmd which are available in the FLYFACTORSURVEY database [24]. Equation 6 suggests that PWM1 has a *λ* of 1.6, and we are trying to find a *λ* for PWM2. Subfigure **c** is a heatmap of the residence time distribution of PWM2 for different values of *λ* and different binding site strength level. Each column of the heatmap represents a specific *λ* value and each row represents a specific binding site strength level measured by the −*l* *o* *g*_10_ of the corresponding top quantiles from low affinity to high affinity sites. Blank regions in the heatmap indicate *λ* values which lead to residence time out of the reference scale that is an indication of unsuitable *λ* values. **d**, **e** and **f** show the correlation of residence time between PWM1 and PWM2 using specific *λ* values of 0.8, 1.4 and 2.0, respectively. The curve in Subfigure E has the lowest mean square error, and so we assign PWM2 to have a *λ*=1.4

The procedure to compute a suitable *λ* is based on the concept of sequence-specific residence time (Eq. 4), as illustrated in Fig. 5. Initially, a well-characterized *λ* is computed or measured for the first PWM of a particular TF, and then we use this value to derive a *λ* that is appropriate for the second PWM of the same TF. As part of the calculation of the *λ* for the second PWM, Fig. 5 c shows a heatmap of the estimated residence times for a TF named lame duck (lmd) in a particular binding strength quantile, at different values of *λ* (ranging from 0.1 to 3.0 as suggested by both [13] and the range of estimated *λ* using Eq. 6 across different organisms). Both PWMs for the TF come from FlyFactorSurvey database [24], but they are derived from different reports with motif logos shown in Fig. 5 b. Blank regions in the heatmap indicate that the choice of *λ* would generate a residence time outside the range of pre-calculated possible residence times using the first PWM and the existing *λ* value implying that the *λ* values for the second PWM are unsuitable. As shown in the heatmap, blank regions often appear in very low values of *λ*. While if *λ* is too large, the possible residence time range from weak to strong binding sites is often very restricted, meaning high affinity sites cannot be distinguished from low affinity sites efficiently. *λ* values with residence times all within the reference range can be further selected, as specified in Methods. Figure 5 d–f compares the residence time values between two different PWMs, at different values of *λ* for the second PWM. We see that the *λ* in Fig. 5 d and 5 f would not allow for consistent residence times between the two PWMs, but Fig. 5 e does provide consistent results. Therefore, the *λ* adopted in Fig. 5 e is picked up as the suitable value for the second PWM. More examples of residence time heatmaps for converting *λ* between different PWMs are shown in Additional file 8: Figure S7. In order to evaluate the consistency of *λ* estimation between the above method and using Eq. 6, we use the examples of 20 *D.melanogaster* TFs with more than 1 version of PWMs available from different experiments. These PWMs are obtained from the *BioConductor* R package *PWMEnrich.Dmelanogaster.background* [17] and their labels are listed in Additional file 9: Table S2. Since there are only few *λ* available from binding profile fitting, just for the purpose of illustration, the reference values of *λ* were pre-calculated from Eq. 6 instead. New *λ* values for PWMs obtained from other experiments are computed using both methods and they show good consistency with each other (adjusted *R* ^2^ = 0.88, Additional file 10: Figure S8). Converting *λ* between these two PWMs in the opposite direction also show similar results (data not shown). It indicates that both methods provide consistent estimates of *λ*, even though they have different core assumptions.

## Discussion

TF binding site strength estimation using PWM-based methods is essential for modelling TF-DNA interaction in functional genomics; but a proper scaling parameter is needed when using the PWM score to estimate TF binding energy. Therefore, we provide two independent methods for estimating the scaling parameter *λ* in different conditions. The simple Eq. 6 is widely applicable, since it only requires a PWM as input, which is easy to implement compared to methods using fitting to ChIP-seq [13, 14]. Our second method converts a *λ* specific to one PWM into *λ* for a different PWM of the same TF. It is based on the definition of sequence-specific residence time from the facilitated diffusion model of TFs on DNA [16]. This method is particularly useful for converting a previously estimated *λ* into the one associated with a more up-to-date or otherwise alternative PWMs.

These two methods are consistent with one another (Additional file 11: Figure S4) and with previously established methods. For instance, Eq. 6 can also provide very similar results compared with the estimated *λ* from ChIP-seq data fitting [13, 14]. Although our estimates of *λ* are mostly consistent with those estimated by Zabet [14] and Roider [13], it is not possible to robustly compare our *λ* estimates to experimentally derived values at large scale, as this data is simply unavailable. Having more such data would also enable us to adjust currently fixed parameters in our equation for different TF families, such as the top-scoring threshold, instead of assuming a uniform value across all TFs. The consistent value range of *λ* in different organisms calculated by this method provides additional support for the applicability of this simple equation. Moreover, the estimated distribution of *λ* values for different TF families make sense in the light of motif choice for each of the TF families [28]. For example, TFs in the zinc-finger TF super-family, including nuclear receptor zinc-finger and *β*- *β*- *α* zinc-finger families, have more flexible binding motifs, which can suit a wider range of possible binding sites than the helix-turn-helix super-family, which has a more restricted motif consensus sequence [26]. In contrast, some TF families belonging to the same super-family and sharing similar binding domain properties can have a strong similarity in *λ* distribution, *e.g.* homeobox family and forkhead family which both belong to the helix-turn-helix super-family. The two TF families that show the highest average z-score of *λ* values (namely, basic leucine-zipper and helix-loop-helix families) tend to form homodimers and heterodimers, though some TFs in other TF families also tend to dimerise *e.g.* some members in homeobox family. If PWM motifs for either monomers or dimers are available, the corresponding *λ* scores can be roughly estimated following the same procedure using Eq. 6, or we can further use the second method mentioned before to convert *λ* values between different PWMs by the keeping residence time consistent. However, our method only considers TF-DNA interaction, ignoring the effects of TF-TF interactions that could stabilize TF binding.

There are some points that should be noted when using the simple equation method: first, it cannot be applied to very short TF motifs that are less than 6 base pairs in length. Since this method depends on calculating the difference between the PWM score of the 0.1 % highest scoring matches and the maximum score, if the motif is only 5 base pairs in length, the number of possible choices for sequence combination of 5 base pairs is only 1024, then the top 0.1 % highest scoring matches is very likely to be nearly equal to the maximum score. However, most eukaryotic motifs are more than 6 base pairs long. Eukaryotic TFs on average cover 15 bp of DNA with a core motif length of 8-15 bp [8]. Thus, this limitation should not be a problem in the majority of cases. However, if a higher threshold *e.g.* top 1 ·10^−5^ is applied with certain adjustment for average mismatch bits in the denominator, it requires the PWM motif to be at least 10 bp long, which will limit the applicability of this simple method. The default cut-off threshold for binding sites is the top 0.1 % of the highest scoring matches, but varying the threshold up to the top 0.001 % does not significantly influence the rank of *λ* (Additional file 2: Figure S5). Note in Eq. 6, the average mismatch energy bit score in the denominator is the one corresponding to the certain top PWM matches threshold, which means if a new threshold is adopted, the average mismatch energy bit score should be updated accordingly, but given very limited binding energy measurement data, it is difficult to select specific values for each corresponding binding site strength level. Thus, we simply compared the rank correlation of *λ*, which is not affected by the linear scaling factor of average mismatch energy bits. Although we estimate *λ* by top scoring genomic sequences, it will not substantially affect the analysis if this is done on random sequences with the same GC content, since given the size of the genome, local binding site patterns will not have much influence on the general distribution of binding site strength. Additional file 1: Figure S6 shows that the number of unique k-mers passing the 0.1 % top scoring matches threshold in genomic sequences correlates well with that in random sequences of the same GC content.

Another assumption in this method is that the mismatch energy tolerance range measured in bits is proportional to the information content of the PWM. This assumption can deal with the bias from the differences in information content of most PWMs; however, it might not hold for PWMs with extremely high information content. For example, the yeast transcription factor IXR1 has an information content of 47 bits according to the PFM from JASPAR [5], which is substantially larger than the average information content of 13.2 bits. In that case, the binding energy will probably be overestimated, which leads to a lower *λ*, but these cases are very rare and only 7 PWMs in our analysis (less than 1.5 %) have information content greater than 20. Further, we note that the experiment by Maerkl et al. [15] was applied only to TFs belonging to the bHLH family. In the absence of any alternative data, we simply assume that this value is scaled by the information content of the PWM; although if more in-vitro binding energy measurements should become available in the future, we suggest adjusting the specific top score threshold and corresponding average mismatch energy bits accordingly.

There are two limitations of this method, which can potentially lead to some biases between different organisms and different TF families. One limitation is related to the calculation of mismatch energy tolerance in different groups of TF families. We apply a single cut-off threshold of the top 0.1 % highest scoring matches for weak binding sites suggested by Wunderlich et al. [21], but it could be possible that for different TF families, different thresholds should be used due to variations in their DNA binding domains. However, it is difficult to choose specific thresholds for every TF family based on the currently available data. Further, from the definition of information content of the PWM, it sums up information content gain from each nucleotide [20]. It implies that longer motifs including more flanking base pairs will have higher information content compared to the shorter ones with only core motifs, which is an artefact of computation. However, there is no satisfactory way to deal with this problem. One possible solution is using the information content per nucleotide instead of the total information content, but this may be problematic as the information content contributed by flanking sequences constitutes only a very small fraction compared to core motifs. Thus, if dividing total information content by the length of the motif, the dilution of information content could lead to even larger biases. Therefore, instead, in our analysis of comparing *λ* value distribution across different organisms and TF families, we control for motif length by normalizing it to the mean in each motif length bin. Another potential solution is trying to define a core motif from one PWM, but this requires detailed knowledge about the TF of interest. Additionally, *λ* will not be a reliable measure of the biochemical stickiness of the TF to the DNA if the PWM itself is not an accurate representation of TF binding. A PWM assumes that each nucleotide position independently contributes to TF binding affinity, which may not be the case [29, 30]. For instance, a study by Storm et al. [31] used both a single nucleotide model and a di-nucleotide model to fit the binding energy measurements [15]. Although they found that the di-nucleotide model provides a better fit to the experimental data, the single nucleotide model could also perform well when non-specific binding energy was taken into account. In addition, the composition of the position frequency matrix of the PWM may contain biases due to the difficulties of attaining an unbiased validated binding site set. Nevertheless, *λ* can give us insights about DNA binding properties of TFs.

Also, it should be pointed out that residence time in this paper refers to an estimate based on biophysical models [10, 16]. However, other papers report inconsistent scales of residence time according to different experimental approaches. For example, the residence time estimations obtained by Competition-ChIP methods [32] do not share the same order of magnitude compared to the residence times measured by FRAP or single molecular tracking [22, 33, 34], which can probably be an artifact of experimental methods or alternatively, the range of residence time truly varies greatly across different TFs [35]. Because the experimentally determined values are not comparable to each other, we simply adopt bioinformatics-based approaches to compute residence time. Since our method converts *λ* between different PWMs of the same TF under the concept of residence time, it avoids fitting inconsistent experimental observations and potential variations in DNA-binding kinetics for different TFs.

Although in many cases PWMs are not optimal representations of binding motifs, they have become almost universally adopted to identify potential TF binding sites. It is important to remember that the value of a PWM score is not directly correlated to the binding energy, but rather depends on the scaling parameter *λ*. Previously, researchers either assumed that *λ* has similar values across different PWMs or estimated it through computationally intensive binding profile fitting methods [13, 14]. There are several alternative ways to identify potential binding sites based on the p-value of the PWM score [12]. Other studies provide tools to combine more local information *e.g.* DNA sequence conservation and epigenetic marks with PWMs to identify potential binding sites with higher confidence [36]. These methods are useful in defining potential binding sites, but their results are difficult to interpret in terms of TF binding energy which is widely used in modeling TF binding dynamics and enhancer activity [8]. Here we provide two simple strategies for estimating *λ*, which will let us more clearly link PWM scores with the energetics of TF binding.

## Conclusion

Using PWMs as representations of binding motifs have become widely adopted to identify potential TF binding sites. It is important to remember that the value of a PWM score is not directly correlated to the binding energy, but rather depends on the scaling parameter *λ*. Previously, researchers either assumed that *λ* has similar values across different PWMs or estimated it through computationally intensive binding profile fitting methods [13, 14]. There are several alternative ways to identify potential binding sites based on the p-value of the PWM score [12]. Other studies provide tools to combine more local information *e.g.* DNA sequence conservation and epigenetic marks with PWMs to identify potential binding sites with higher confidence [36]. These methods are useful in defining potential binding sites, but their results are difficult to interpret in terms of TF binding energy, which is widely used in modeling TF binding dynamics and enhancer activity [8]. Here we provide two simple strategies for estimating *λ*, which will let us more clearly link PWM scores with the energetics of TF binding. One approach is to simply apply Eq. 6 to estimate *λ* only based on the given PWM and genome background sequences of a specific organism. It provides results consistent with those estimated by Zabet [14] and Roider [13] in most cases, though there is a small number of exceptions. Further, *λ* value distribution for different TF families from this method are consistent with DNA binding properties of TF families [26, 28], which further supports the applicability of this simple method. Another approach converts *λ* between two PWMs of the same TF based on the consistency of residence times. It is useful when we get alternative versions of PWMs from different databases and want to estimate binding site strength in a consistent manner. Both of the approaches we developed are much compute-efficient than previous methods of TF binding profile fitting [13, 14].

## Additional files

Additional file 1
**Figure S6.** Comparison of unique k-mer number passing 0.1 % top PWM score threshold in genomic background versus that in random sequences. For each TF PWM motif, we calculated the logarithm of the number of unique k-mers that passes the threshold in both genomic background and random sequences that have the same GC content and they correlate well with adjusted *R* ^2^ equals 0.98, p-value <10^−16^. (PDF 14.8KB)

Additional file 2
**Figure S5.** Correlation of *λ* rank obtained by using different top score thresholds in Eq. 6. We compare the *λ* rank for different TFs in each group of organisms (subfigure A, B for *S. cerevisiae*, C and D for *D. melanogaster*, E and F for vertebrate PWM motifs) by adopting a different top score threshold of top 0.01 % or 0.001 % instead of the default value of 0.1 % in Eq. 6. The adjusted *R* ^2^ for the *λ* rank correlation between 0.1 % and 0.01 % thresholds for *S. cerevisiae*, *D. melanogaster*, and vertebrate motifs are 0.94, 0.89 and 0.80, respectively, with p-values all less than 10^−8^. As for the *λ*rank correlation between 0.1 % and 0.001 % thresholds, the adjusted *R* ^2^ are 0.87, 0.92 and 0.74, respectively (p-values all less than 10^−6^). (PDF 33.2KB)

Additional file 3
**Table S3.** (TXT 0.232KB)

Additional file 4
**Figure S3.** Estimated *λ* distribution in relation to PWM motif length. Each color coded point represents a specific *λ* value of a TF estimated by Eq. 6 for *S. cerevisiae* (green), *D. melanogaster* (red), and vertebrate (blue).There is a positive correlation between estimated *λ* value and TF motif length with adjusted *R* ^2^ equals 0.33. (PDF 22.6KB)

Additional file 5
**Figure S1.** The relationship between maximum PWM score and information content of PWMs. Individual dots represents each PWM generated from the non-redundant PFM JASPAR-CORE database [5] after the filtering procedures specified in the Methods section. There is a strong positive correlation between the information content of the PWM and the maximum possible PWM score that could be generated by that PWM, with an adjusted *R* ^2^ value of 0.597. (PDF 25.9KB)

Additional file 6
**Table S1.** (TXT 12.7KB)

Additional file 7
**Figure S2.** Estimated *λ*distribution across major TF families. BBA-ZF represents the *λ*distribution for *β*- *β*- *α* zinc-finger family; NR is zinc-finger nuclear receptor family; L-zipper stands for the basic leucine-zipper family; HLH is helix-loop-helix family; Homeo is homeobox family; FK is fork-head family and HMG is high mobility group family. For each group, *λ* was calculated by Eq. 6. (PDF 5.33KB)

Additional file 8
**Figure S7.** Heatmaps for *λ* conversion between different PWMs. These are additional examples of heatmaps of sequence-specific residence time that are used for *λ* conversion between different PWMs of the same TF. Alternative versions of PWMs are from *BioConductor* R package of *PWMEnrich.Dmelanogaster.background* [17]. Each column of the heatmaps represents a specific *λ* value and each row represents a specific binding site strength level. (PDF 25.5KB)

Additional file 9
**Table S2.** (TXT 1.015KB)

Additional file 10
**Figure S8.** Consistency of *λ* estimation between two methods. This figure shows the correlation between *λ* values obtained from Eq. 6 and from *λ* conversion using the heatmap of sequence-specific residence time. The adjusted *R* ^2^ is 0.88, p-value =5.9·10^−5^. (PDF 11.1KB)

Additional file 11
**Figure S4.** Comparison of *λ* values calculated by using different pseudo-count values in PWMs. Subfigure A shows the comparison between the *λ*values obtained by using PWMs with pseudocounts of 1 and 3 (the adjusted *R* ^2^ is 0.973), while subfigure B compares pseudocounts of 1 and 0.3 (the adjusted *R* ^2^ is 0.978). Each dot represents a TF from 100 randomly chosen vertebrate TFs in JASPAR database [5]. (PDF 31.2KB)
