# Supplementary figures and images for: Reliable scaling of position weight matrices for binding strength comparisons between transcription factors
Source: BMC Bioinformatics. 2015 Aug 20;16:265. doi: 10.1186/s12859-015-0666-1 (PMC4545934; doi:10.1186/s12859-015-0666-1)

log(number of unique k-mers) in genome background

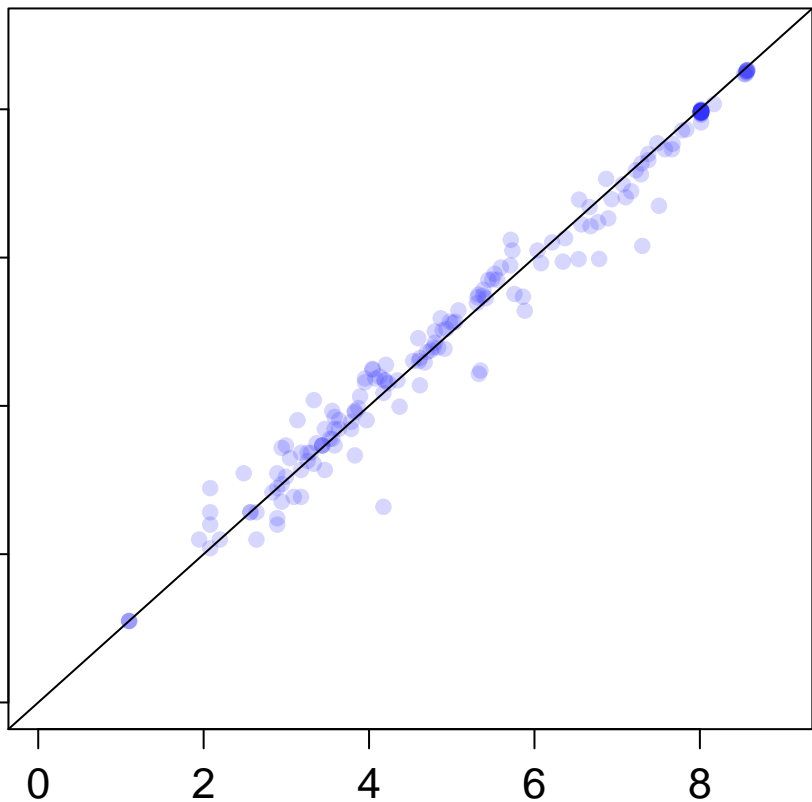

log(number of unique k-mers) in random sequences

Supplement: Additional file 1 — Figure S6. Comparison of unique k-mer number passing 0.1 % top PWM score threshold in genomic background versus that in random sequences. For each TF PWM motif, we calculated the logarithm of the number of unique k-mers that passes the threshold in both genomic background and random sequences that have the same GC content and they correlate well with adjusted R 2 equals 0.98, p-value <10−16. (PDF 14.8KB) [file 12859_2015_666_MOESM1_ESM.pdf]

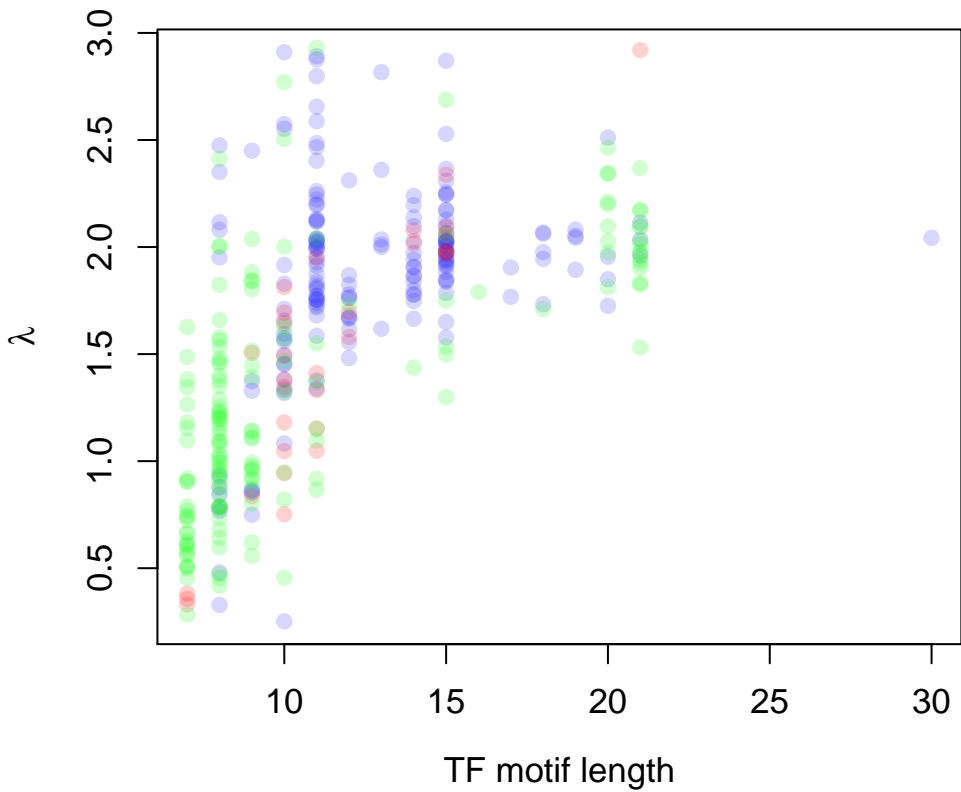

Supplement: Additional file 4 — Figure S3. Estimated λ distribution in relation to PWM motif length. Each color coded point represents a specific λ value of a TF estimated by Eq. 6 for S. cerevisiae (green), D. melanogaster (red), and vertebrate (blue).There is a positive correlation between estimated λ value and TF motif length with adjusted R 2 equals 0.33. (PDF 22.6KB) [file 12859_2015_666_MOESM4_ESM.pdf]

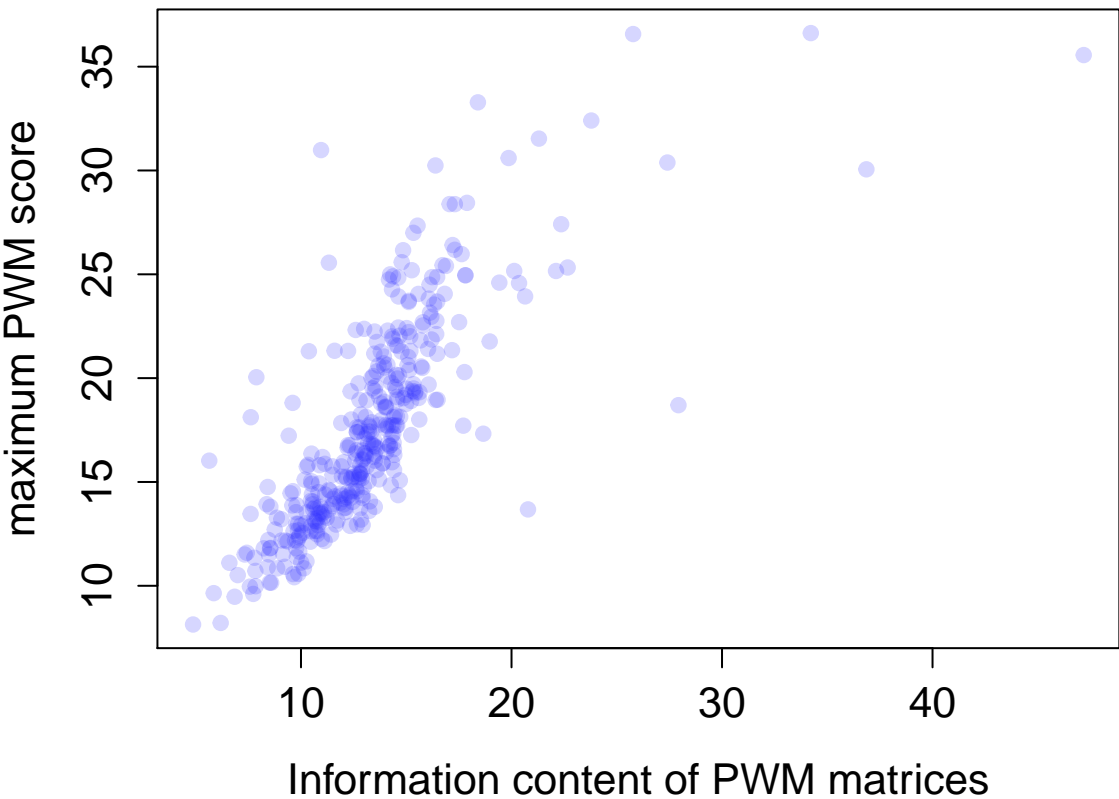

Supplement: Additional file 5 — Figure S1. The relationship between maximum PWM score and information content of PWMs. Individual dots represents each PWM generated from the non-redundant PFM JASPAR-CORE database [5] after the filtering procedures specified in the Methods section. There is a strong positive correlation between the information content of the PWM and the maximum possible PWM score that could be generated by that PWM, with an adjusted R 2 value of 0.597. (PDF 25.9KB) [file 12859_2015_666_MOESM5_ESM.pdf]

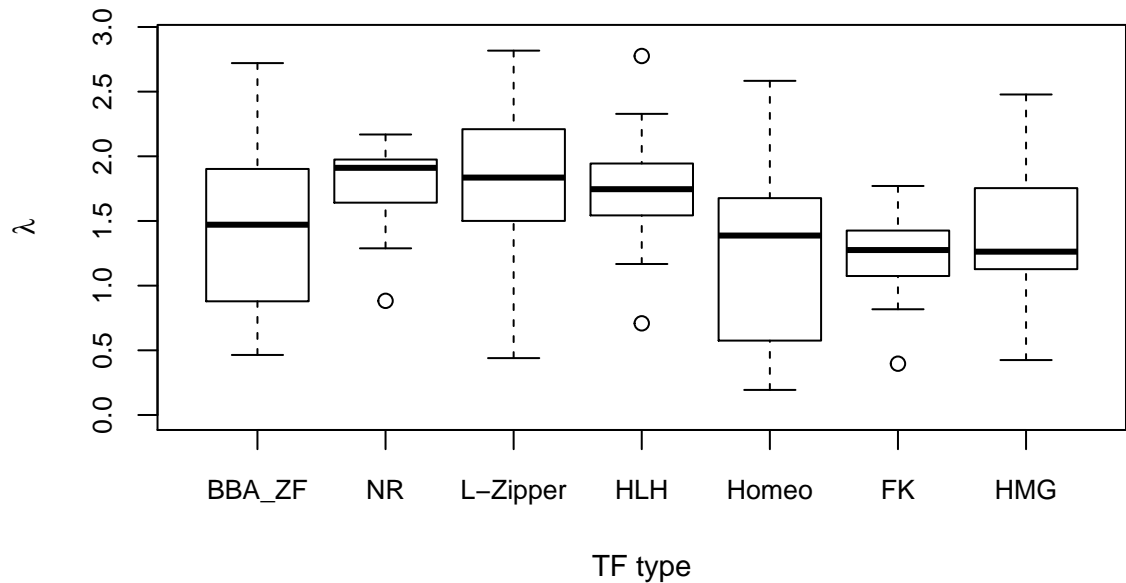

Supplement: Additional file 7 — Figure S2. Estimated λdistribution across major TF families. BBA-ZF represents the λdistribution for β- β- α zinc-finger family; NR is zinc-finger nuclear receptor family; L-zipper stands for the basic leucine-zipper family; HLH is helix-loop-helix family; Homeo is homeobox family; FK is fork-head family and HMG is high mobility group family. For each group, λ was calculated by Eq. 6. (PDF 5.33KB) [file 12859_2015_666_MOESM7_ESM.pdf]

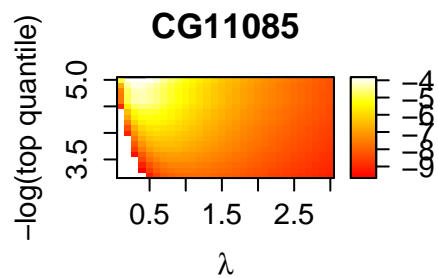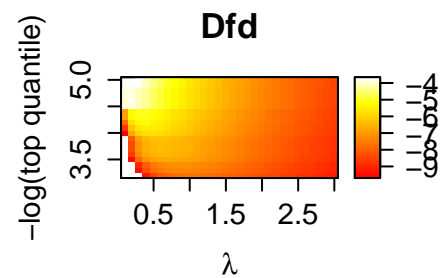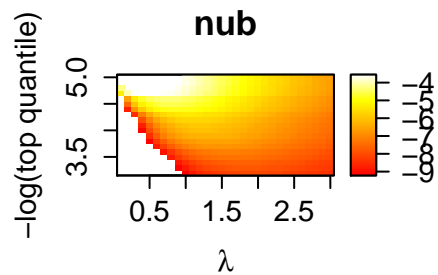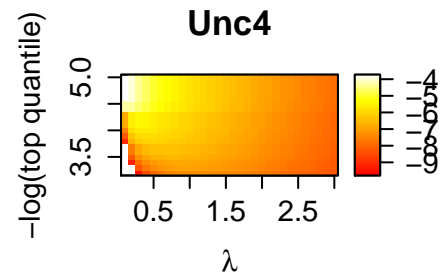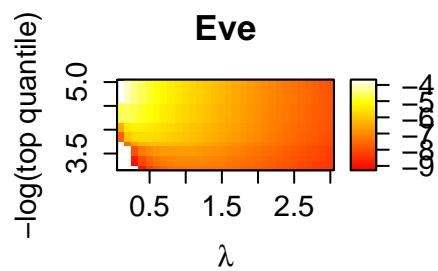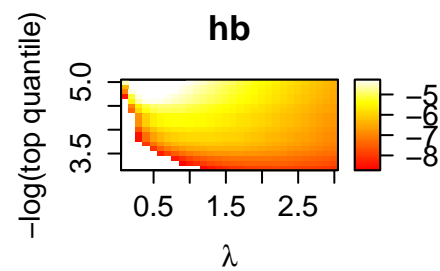

Supplement: Additional file 8 — Figure S7. Heatmaps for λ conversion between different PWMs. These are additional examples of heatmaps of sequence-specific residence time that are used for λ conversion between different PWMs of the same TF. Alternative versions of PWMs are from BioConductor R package of PWMEnrich.Dmelanogaster.background [17]. Each column of the heatmaps represents a specific λ value and each row represents a specific binding site strength level. (PDF 25.5KB) [file 12859_2015_666_MOESM8_ESM.pdf]

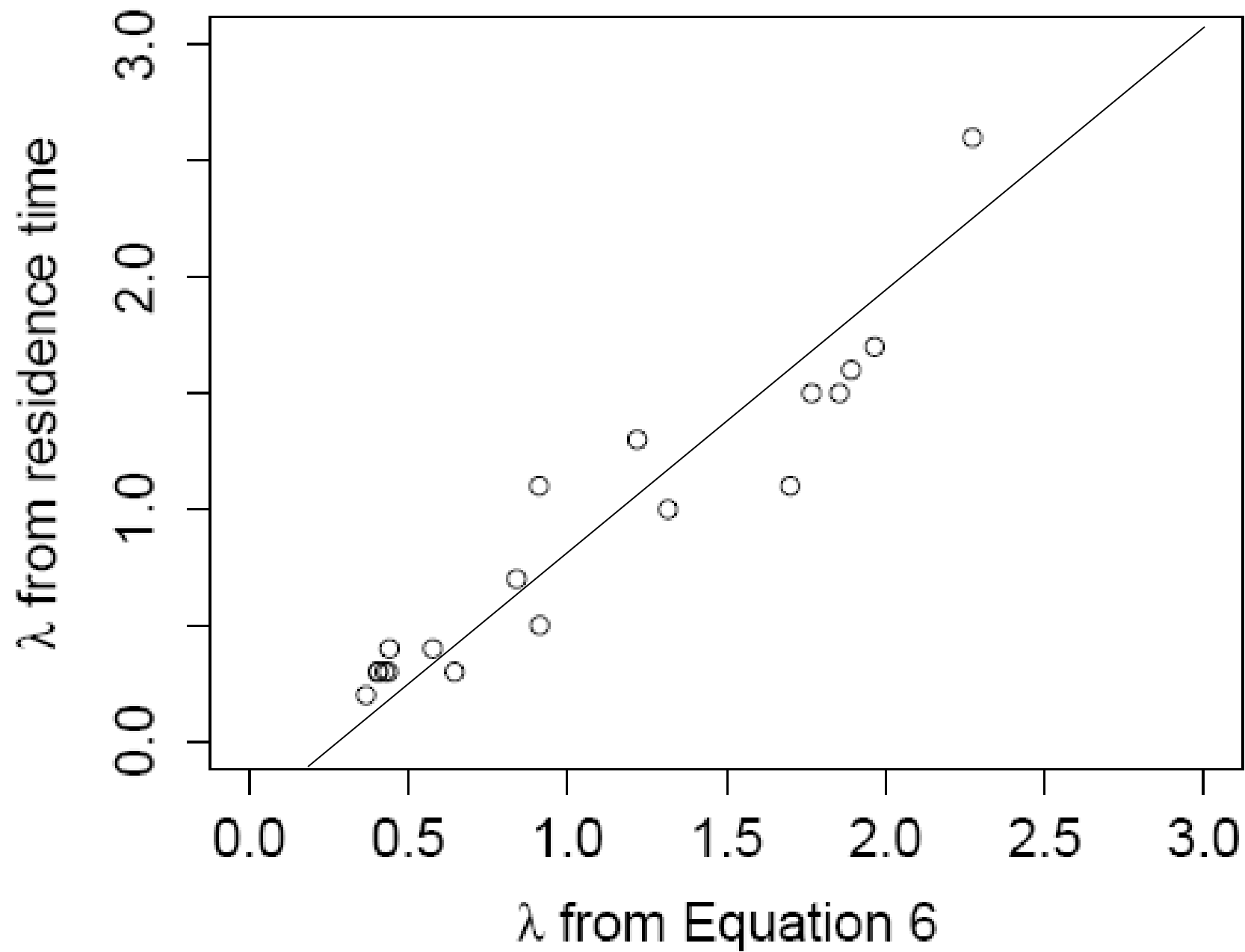

Supplement: Additional file 10 — Figure S8. Consistency of λ estimation between two methods. This figure shows the correlation between λ values obtained from Eq. 6 and from λ conversion using the heatmap of sequence-specific residence time. The adjusted R 2 is 0.88, p-value =5.9·10−5. (PDF 11.1KB) [file 12859_2015_666_MOESM10_ESM.pdf]

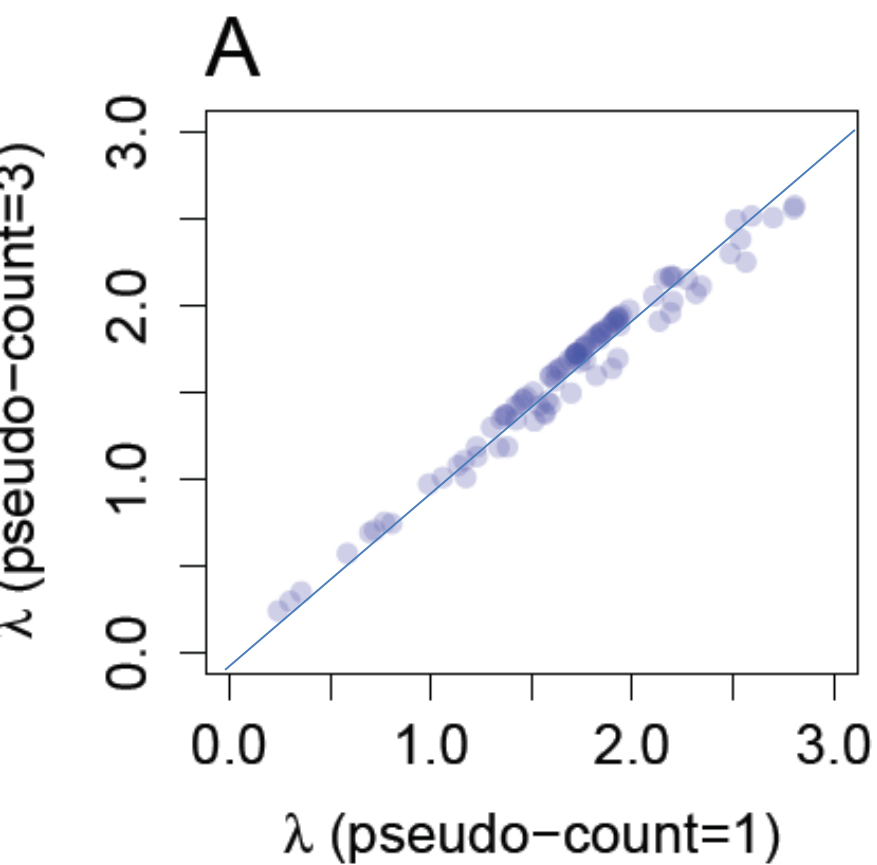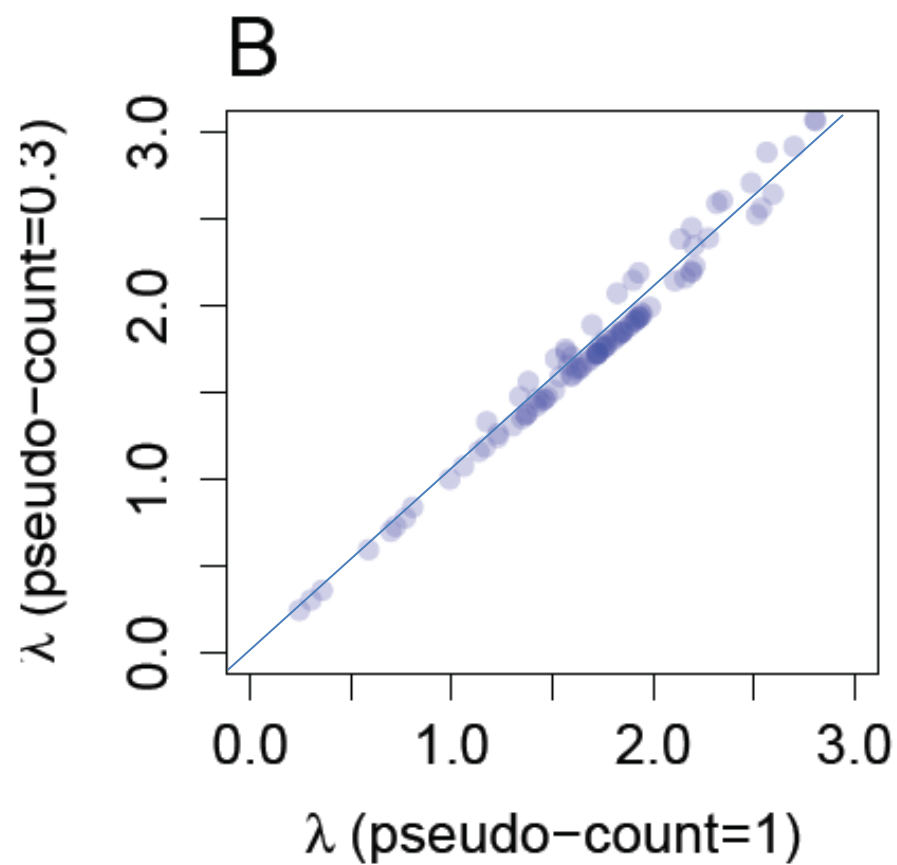

Supplement: Additional file 11 — Figure S4. Comparison of λ values calculated by using different pseudo-count values in PWMs. Subfigure A shows the comparison between the λvalues obtained by using PWMs with pseudocounts of 1 and 3 (the adjusted R 2 is 0.973), while subfigure B compares pseudocounts of 1 and 0.3 (the adjusted R 2 is 0.978). Each dot represents a TF from 100 randomly chosen vertebrate TFs in JASPAR database [5]. (PDF 31.2KB) [file 12859_2015_666_MOESM11_ESM.pdf]
