# Supplementary material for: Reliable scaling of position weight matrices for binding strength comparisons between transcription factors
Source: BMC Bioinformatics. 2015 Aug 20;16:265. doi: 10.1186/s12859-015-0666-1 (PMC4545934; doi:10.1186/s12859-015-0666-1)
Supplement: Additional file 2 — Figure S5. Correlation of λ rank obtained by using different top score thresholds in Eq. 6. We compare the λ rank for different TFs in each group of organisms (subfigure A, B for S. cerevisiae, C and D for D. melanogaster, E and F for vertebrate PWM motifs) by adopting a different top score threshold of top 0.01 % or 0.001 % instead of the default value of 0.1 % in Eq. 6. The adjusted R 2 for the λ rank correlation between 0.1 % and 0.01 % thresholds for S. cerevisiae, D. melanogaster, and vertebrate motifs are 0.94, 0.89 and 0.80, respectively, with p-values all less than 10−8. As for the λrank correlation between 0.1 % and 0.001 % thresholds, the adjusted R 2 are 0.87, 0.92 and 0.74, respectively (p-values all less than 10−6). (PDF 33.2KB) [file 12859_2015_666_MOESM2_ESM.pdf]

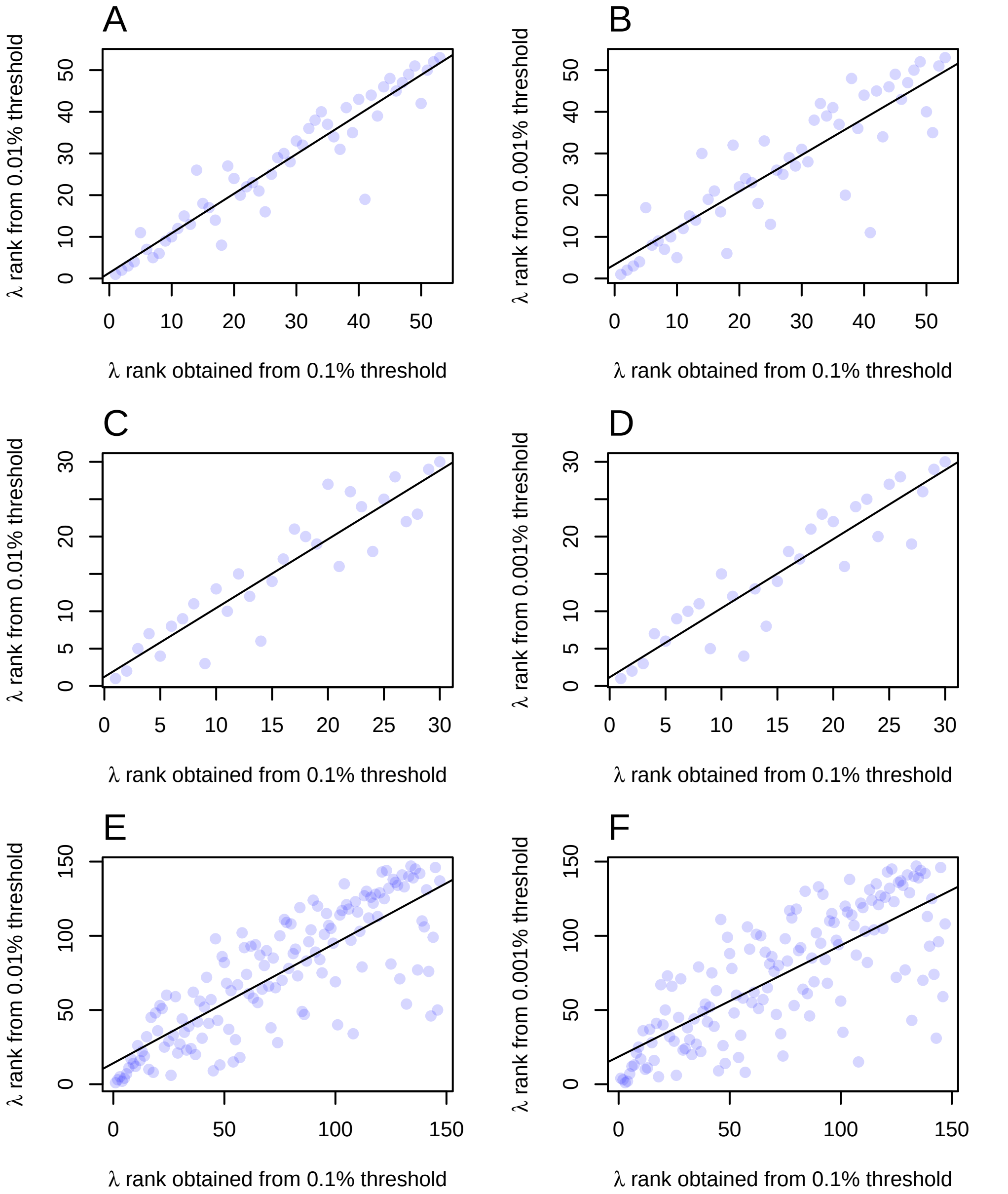
A
B
C
D
E
F
λ rank from 0.01% threshold
λ rank from 0.001% threshold
λ rank obtained from 0.1% threshold
